# The Dinoflagellate Toxin 20-Methyl Spirolide-G Potently Blocks Skeletal Muscle and Neuronal Nicotinic Acetylcholine Receptors

**DOI:** 10.3390/toxins8090249

**Published:** 2016-08-24

**Authors:** Aurélie Couesnon, Rómulo Aráoz, Bogdan I. Iorga, Evelyne Benoit, Morgane Reynaud, Denis Servent, Jordi Molgó

**Affiliations:** 1 Institut des Neurosciences Paris-Saclay, UMR 9197 CNRS/Université Paris-Sud, F-91190 Gif-sur-Yvette, France; aurelie.couesnon@inaf.cnrs-gif.fr (A.C.); romulo.araoz@cea.fr (R.A.); evelyne.benoit@cea.fr (E.B.); 2 Commissariat à l'énergie atomique et aux énergies alternatives (CEA), Institut de Biologie et Technologies de Saclay (IBITECS), Université Paris-Saclay, Service d’Ingénierie Moléculaire des Protéines, bâtiment 152, F-91191 Gif-sur-Yvette, France; morgane.reynaud@hotmail.fr (M.R.); denis.servent@cea.fr (D.S.); 3 Centre National de la Recherche Scientifique (CNRS), Institut de Chimie des Substances Naturelles, UPR 2301, Labex LERMIT, F-91198 Gif-sur-Yvette, France; bogdan.iorga@cnrs.fr

**Keywords:** dinoflagellate toxin, spirolides, nicotinic acetylcholine receptors, neuromuscular transmission, *Xenopus* oocytes, nicotinic currents, competition-binding assays, molecular docking

## Abstract

The cyclic imine toxin 20-methyl spirolide G (20-meSPX-G), produced by the toxigenic dinoflagellate *Alexandrium ostenfeldii*/*Alexandrium peruvianum*, has been previously reported to contaminate shellfish in various European coastal locations, as revealed by mouse toxicity bioassay. The aim of the present study was to determine its toxicological profile and its molecular target selectivity. 20-meSPX-G blocked nerve-evoked isometric contractions in isolated mouse neuromuscular preparations, while it had no action on contractions elicited by direct electrical stimulation, and reduced reversibly nerve-evoked compound muscle action potential amplitudes in anesthetized mice. Voltage-clamp recordings in *Xenopus* oocytes revealed that 20-meSPX-G potently inhibited currents evoked by ACh on *Torpedo* muscle-type and human α7 nicotinic acetylcholine receptors (nAChR), whereas lower potency was observed in human α4β2 nAChR. Competition-binding assays showed that 20-meSPX-G fully displaced [^3^H]epibatidine binding to HEK-293 cells expressing the human α3β2 (Ki = 0.040 nM), whereas a 90-fold lower affinity was detected in human α4β2 nAChR. The spirolide displaced [^125^I]α-bungarotoxin binding to *Torpedo* membranes (Ki = 0.028 nM) and in HEK-293 cells expressing chick chimeric α7-5HT_3_ nAChR (Ki = 0.11 nM). In conclusion, this is the first study to demonstrate that 20-meSPX-G is a potent antagonist of nAChRs, and its subtype selectivity is discussed on the basis of molecular docking models.

## 1. Introduction

**Figure 1:**
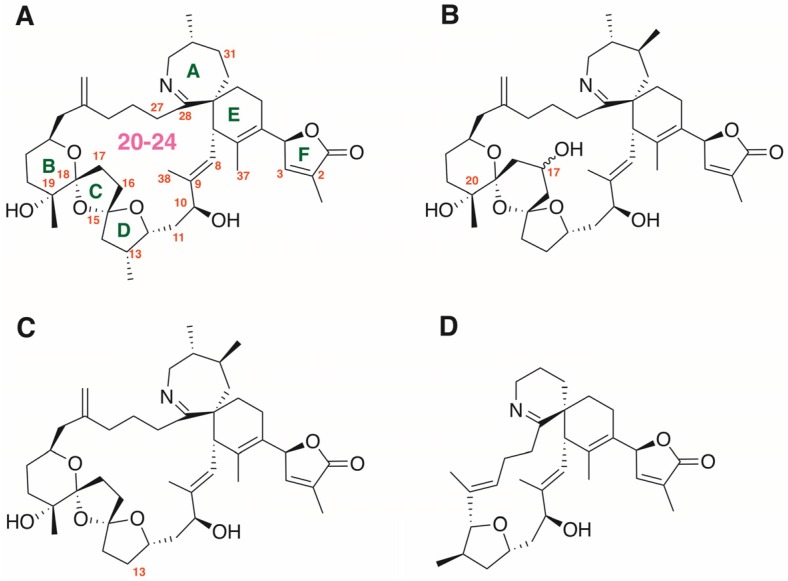
(**A**) Chemical structure of spirolide A toxin (the first member of the spirolide family described). The positions at which variations in the substitution pattern have been described are numbered, and the size of the macrocyclic is shown in magenta; (**B**) chemical structure of 20-methyl spirolide G.; (**C**) chemical structure of 13-desmethyl spirolide C; and (**D**) chemical structure of gymnodimine A.

Spirolides were found in the 1990s in Canada [1,2], when uncommon mouse toxicities were detected with scallop and mussel extracts. Since then, spirolides were shown to have an outstanding diversity in chemical profiles and a world-wide distribution. Spirolides have their place in the cyclic imine group of neurotoxins, which also include prorocentrolides, spiro-prorocentrimine, gymnodimines, pinnatoxins, pteriatoxins, and portimine (for recent reviews see [3,4,5]). At present, sixteen structurally-related spirolides sharing common scaffolds have been reported. The general chemical structure of spirolides is characterized by a macrocycle (with a ring size between 20 and 24, as shown in Figure 1), a seven-membered cyclic imine group, which constitutes a key pharmacophore element, and the spiroketal cyclic, too, which can be 6,5,5-spiroketal (spirolides A–F), 6,5-spiroketal (spirolides H and I), or 6,6,5-spiroketal in the case of spirolide G (Figure 1). The cyclic imine moiety is responsible for the toxicity of these compounds, since spirolides E and F, which have an acyclic aminoketone, exhibit no toxicity [2].

The production of spirolides has been so far associated to the dinoflagellates *Alexandrium ostenfeldii* [6,7] and the closely related species *Alexandrium peruvianum* [8]. Important variations in the chemical profile of spirolides have been reported for strains identified in distinct coastal regions [9,10]. 

In the North Sea, 20-methyl spirolide-G (20-meSPX-G) was the main spirolide toxin that was detected and identified both in shellfish and plankton samples from Sognefjord in Norway [11]. Later, fatty acid acyl esters of 20-meSPX-G were also detected by LC/MS in shellfish samples from Norway, but not in algal samples, indicating that 20-meSPX-G, through the action of acyl transferases, can be esterified by shellfish metabolism [12]. The 20-meSPX-G was also found in contaminated Norwegian blue mussels [13], and on isolates of *A.* *ostenfeldii* from Limfjorden in Denmark [14] and from the Scottish North Sea [15]. In contrast, *A. ostenfeldii* strains from the Baltic Sea were reported producing paralytic shellfish toxins, but not spirolide toxins [16].

The acute toxicity of spirolides, appraised by mouse bioassays, classes them as “fast-acting” toxins, because they induced rapid onset of neurological symptoms previous to death by respiratory arrest within tens of minutes following intraperitoneal/oral administration of lethal doses of these toxins [17]. Among spirolide congeners, 20-meSPX-G is considered as one of the most toxic when administered by intraperitoneal (i.p.) injection (mouse LD_50_ = 0.008 mg·kg^−1^), while is less active when given orally by gavage (mouse LD_50_ = 0.160 mg·kg^−1^) or by feeding (mouse LD_50_ = 0.630 mg·kg^−1^) [18]. Nevertheless, a lower acute toxicity was described in another study reporting the absence of lethality in mice after an i.p. administration of 0.063 mg·kg^−1^ of 20-meSPX-G [19]. 

Previous studies have unequivocally established that compounds belonging to the so-called cyclic imine toxin family like gymnodimine A [20] 13-desmethyl spirolide C [21,22], 13,19-didesmethyl spirolide C [23], pinnatoxins A and G [24,25] and pinnatoxins E, F and G [26] are potent antagonists of nicotinic acetylcholine receptors (nAChRs). 

To the best of our knowledge, there have been no studies regarding the interaction of 20-meSPX-G with muscle-type and neuronal nAChR subtypes. Therefore, the aim of the present study was to determine, using both functional electrophysiological and competition-binding studies, the toxicological action of 20-meSPX-G on *Torpedo* (α1_2_β1γδ) and mouse (α1_2_β1δε) muscle-type nAChRs, as well as the affinity for neuronal chick chimeric α7-5HT_3_, human α3β2, and human α4β2 nAChRs, and to define the rank order of potency of 20-meSPX-G against the distinct nAChR subtypes. In addition, a structural explanation is proposed, based on molecular computational modeling for 20-meSPX-G-nAChRs interactions.

## 2. Results

### 2.1. Block of Nerve-Evoked Muscle Contraction

**Figure 2:**
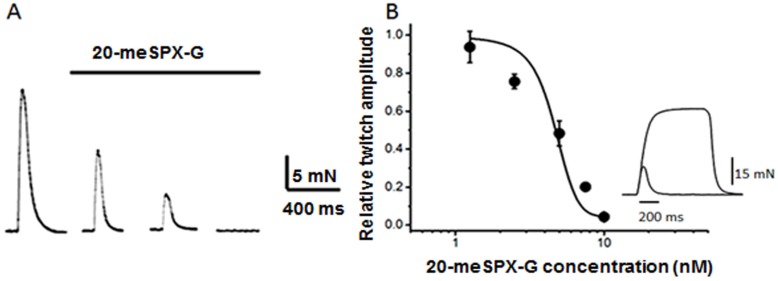
(**A**) Nerve-evoked isometric twitch responses on isolated mouse extensor digitorum longus (EDL) before and after the action of 10 nM 20-meSPX-G; and (**B**) concentration-response curve for the action of 20-meSPX-G on nerve-evoked isometric twitch response. Data points represent the normalized twitch response, relative to the respective controls. Each point is the mean ± SEM of 4 nerve muscle preparations at 60 min toxin exposure. The inset in **B** shows an example of superimposed twitch and tetanus response (40 Hz) triggered by direct electrical muscle stimulation when the nerve-evoked twitch was completely blocked by 10 nM 20-meSPX-G.

Exposure of isolated mouse extensor digitorum longus (EDL) neuromuscular preparations, endowed with the α1_2_βδε nAChR, to the action of 20-meSPX-G revealed that the spirolide blocked in a time- and concentration-dependent manner the isometric twitch responses elicited by motor nerve stimulation (Figure 2). The onset of the contraction block was rapid at high 20-meSPX-G concentration, with a complete block of the twitch response within 60 min at concentrations above 10 nM.

The block of neuromuscular transmission by 20-meSPX-G was not completely reversed by continuous wash-out for 30 min with a spirolide-free medium. At the time neuromuscular transmission was blocked, direct electrical stimulation of the EDL muscle evoked isometric twitch contractions (Figure 2B, inset), indicating a lack of a direct effect on the muscle contractile machinery.

### 2.2. Local Neuromuscular Block in Vivo

**Figure 3:**
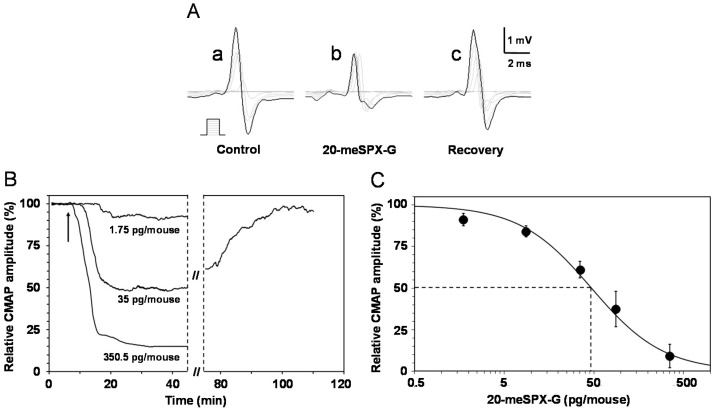
Effects of i.m. local injections of 20-meSPX-G in vivo on the mouse neuromuscular system. (**A**) Compound muscle action potentials (CMAPs) recorded from the tail muscle in response to caudal motor nerve stimulation (increasing intensities, scheme), before and at various times after injection of the cyclic imine toxin (35 pg/mouse); (**B**) time-course of the effects of 20-meSPX-G injections (1.75, 35 and 350.5 pg/mouse) on the CMAP maximal amplitude. Arrows indicate the time of injections; and (**C**) dose-response curve of the effects of 20-meSPX-G injections on the maximal CMAP amplitude. Each value represents the mean ± SEM of data obtained from 4–6 mice, and is expressed relatively to that obtained before injections. The sigmoid curve was obtained by nonlinear regression analysis through data points (*R*^2^ ≥ 0.99). The 20-meSPX-G dose required to block 50% of the maximal CMAP amplitude (dashed lines) was 47 pg/mouse.

An in vivo study of the excitability properties of the mouse neuromuscular system after i.m. injection of 20-meSPX-G (from 1.75 to 350.5 pg/mouse) revealed that the major effect of the toxin was a noticeable time- and dose-dependent reduction of the compound muscle action potential (CMAP) amplitude in response to motor nerve stimulation, as shown in Figure 3A. 

The onset of the effect depended on the 20-meSPX-G dose and ranged from 15.8 ± 1.9 min (*n* = 4 mice) for the lowest dose used (1.75 pg/mouse) that produced a mean 9% block to 7.7 ± 2.1 min (*n* = 4 mice) for the highest dose (350.5 pg/mouse) used that blocked 91% of the CMAP amplitude. Interestingly, the blocking effect of 20-meSPX-G was spontaneously partially reversed, since the CMAP maximal amplitude in the presence of 35–87.6 pg/mouse of toxin returned to 78.5% ± 15.6% of controls within 53.3 ± 19.6 min (*n* = 6 mice), as shown in a typical experiment (Figure 3B). No significant action of the solvent used to dilute the toxin (i.e., ≥1% methanol) was observed on the CMAP amplitude when compared to controls (see also [27]). This indicates that the vehicle used to dissolve 20-meSPX-G had, by itself, no discernable effect on maximal CMAP amplitude.

A clear dose-dependent inhibition of the maximal CMAP amplitude was determined as a function of the 20-meSPX-G doses injected, as shown in Figure 3C. Under these conditions, the effective dose of 20-meSPX-G required to block 50% of the maximal CMAP amplitude (ED_50_) was calculated, giving a value of 47 pg/mouse (*R*^2^ = 0.99), corresponding to 1.7 ng/kg or 2.4 pmol/kg mouse.

### 2.3. Block of Torpedo Muscle-Type nAChR Incorporated to Xenopus Oocytes

**Figure 4:**
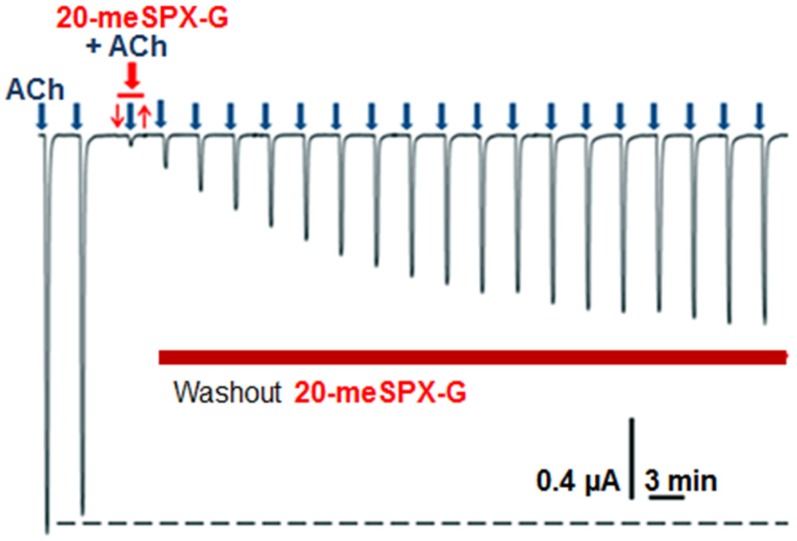
Activation of *Torpedo* α1_2_β1γδ nAChR incorporated into the oocyte membrane by an EC_50_ of ACh (25 µM), applied for 15 s duration (blue arrows), the inhibitory action of 3.12 nM 20-meSPX-G, and the washout of the spirolide from the medium. The two first inward nicotinic currents, recorded at −60 mV holding potential, correspond to the control ACh-evoked currents. The red tracing above the current trace denotes the perfusion of 20-meSPX-G before ACh perfusion (indicated by the blue arrow). No current was evoked by the perfusion of the spirolide alone, indicating that it has no direct agonist action on the receptors, while when ACh was applied in the presence of 20-meSPX-G (red arrow) a marked reduction in the ACh-evoked current occurred. The washout of 20-meSPX-G from the medium (indicated by a brown tracing below the current trace) only allowed partial recovery (≥50%) of control ACh-evoked currents.

Studies were carried out on *Xenopus* oocytes having incorporated to their membranes the *Torpedo* muscle-type α1_2_β1γδ nAChR in order to determine whether 20-meSPX-G had an agonist and/or inhibitory action on this receptor subtype. For this, microtransplanted oocytes were voltage-clamped at −60 mV, and ACh was applied at the EC_50_ (25 µM) evoking typical inward nicotinic currents that exhibited similar amplitudes when delivered at 3 min interval and, upon washing, returned to the current basal line, as shown in a typical experiment (Figure 4). The fact that the amplitude of the ACh-evoked current was usually ≥1 µA (*n* = 6) was indicative of a good incorporation of the *Torpedo* muscle-type receptor into the oocyte membrane. However, when 3.1 nM 20-meSPX-G was applied to the same oocyte no change in the basal line current was observed, indicating that the toxin had no agonist action on the *Torpedo* muscle-type α1_2_β1γδ nAChR (Figure 4).

**Figure 5:**
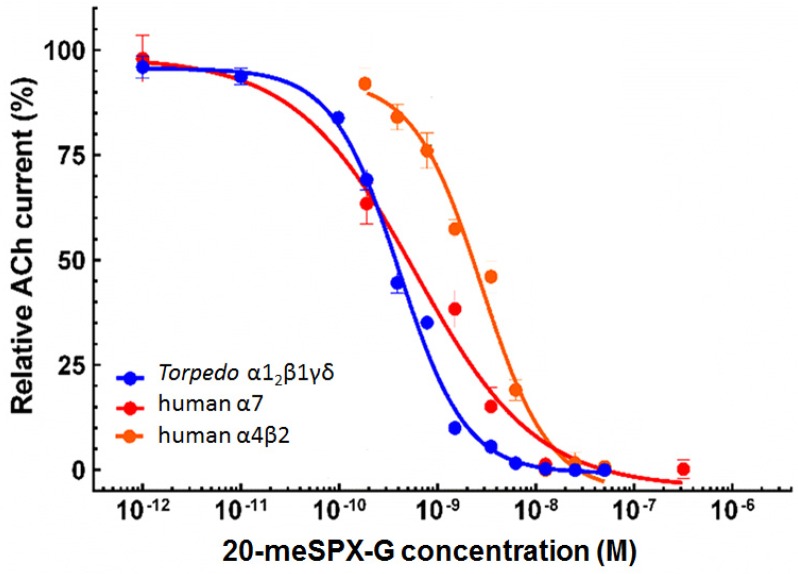
Concentration-dependent inhibition of ACh-elicited nicotinic currents by 20-meSPX-G in *Torpedo* α1_2_β1γδ (blue curve); human α7 (red curve); and human α4β2 (orange curve) nAChRs incorporated or expressed in *Xenopus* oocytes. Peak amplitudes of ACh-evoked currents (mean ± SEM), were recorded under voltage-clamp conditions at −60 mV holding membrane potential, in the presence of 20-meSPX-G were normalized to control currents and fitted to the Hill equation. The concentration of ACh used for each nAChR subtype was the EC_50_ experimentally determined (see text for details).

In contrast, when ACh was applied together with 3.1 nM 20-meSPX-G a marked block of the inward nicotinic current was observed, as shown in Figure 4. This blockade of nAChR was persistent, as determined by washing out 20-meSPX-G from the extracellular medium and delivering a constant ACh concentration spaced by a 3 min interval, since the recovery of the ACh-evoked current did not attain 50% within a 60 min period (Figure 4). These results suggest that 20-meSPX-G has a slow K_off_ from the *Torpedo* α1_2_β1γδ nAChR subtype. As shown in Figure 5, the inhibitory action of 20-meSPX-G on the muscle-type receptor was concentration-dependent, with an IC_50_ = 0.36 nM (0.29−0.45 nM, 95% confidence intervals, 50 oocytes from 8 *Xenopus* donors).

### 2.4. Block of Neuronal nAChRs Expressed in Oocytes

Functional analysis of the action of 20-meSPX-G was also performed using voltage-clamp recordings in *Xenopus* oocytes previously transfected with human α7 or α4β2-encoding cDNAs. The perfusion of 350 μM ACh (EC_50_ for ACh) for 3 s into oocytes, expressing the human α7 nAChR, elicited inward currents that varied in peak amplitudes between 0.8−3 µA at a holding membrane potential of −60 mV (*n* = 30 oocytes tested from five different *Xenopus*). The 20-meSPX-G significantly reduced the peak amplitude of ACh-evoked currents, as shown in the concentration-inhibition curve of Figure 5, with an IC_50_ = 0.48 nM (0.09–2.5 nM, 95% confidence intervals, 30 oocytes from five different *Xenopus* donors).

In *Xenopus* oocytes expressing the human α4β2 nAChR, 20-meSPX-G also blocked the current elicited by 150 μM ACh (EC_50_ for ACh in the α4β2 nAChR subtype), as disclosed in the concentration-inhibition curve shown in Figure 5. The IC_50_ = 2.1 nM (1.4−3.1 nM, 95% confidence intervals, 33 oocytes from six different *Xenopus* female donors), indicates that α4β2 nAChR subtype is less sensitive to the action of 20-meSPX-G than the α7 nAChR.

### 2.5. Binding-Competition Assays between 20-meSPX-G and Radioligands

**Figure 6:**
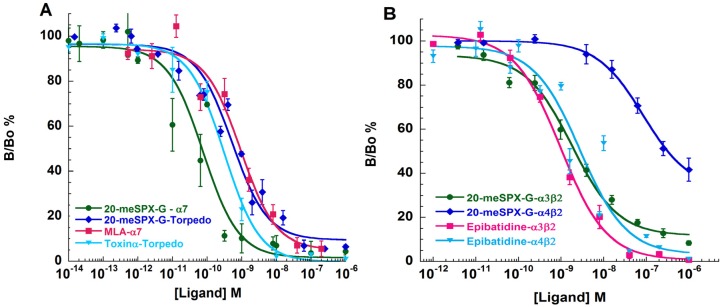
Dose-dependent inhibition of [^125^I]α-BTX binding on *Torpedo* or α7-5HT3 receptors by 20-meSPX-G, α-toxin, and methyllycaconitine (MLA) (**A**) and dose-dependent inhibition of [^3^H]epibatidine binding on α3β2 and α4β2 receptors with 20-meSPX-G and epibatidine (**B**).

To obtain further information on the interaction between 20-meSPX-G and nAChR subtypes concerning their nAChR selectivity profile, binding affinities, and antagonist potencies, competition-binding assays were carried out with purified *Torpedo* membranes expressing muscle-type (α1_2_βγδ) nAChR, and in HEK-293 cells expressing the chimeric chick neuronal α7-5HT_3_, and the human α4β2, and α3β2 nAChR subtypes using both [^125^I]α-bungarotoxin ([^125^I]α-BTX) and [^3^H]epibatidine as radiotracers. As shown in Figure 6A, 20-meSPX-G concentration-dependently displaced [^125^I]α-BTX from *Torpedo* membranes expressing the muscle-type nAChR, and from HEK-293 cells expressing the chimeric α7-5HT_3_ neuronal nAChR. Furthermore, 20-meSPX-G, in the range of concentrations studied, fully displaced [^3^H]epibatidine binding to HEK-293 cells expressing the human α3β2 and α4β2 subtypes (Figure 6B). The affinity constants computed from the binding-competition curves clearly show that 20-meSPX-G interacts with sub-nanomolar affinity with the muscle-type, α3β2, and α7-5HT_3_ nAChRs. On these receptors, 20-meSPX-G was as effective as high-affinity reference-standards, such as the snake α-toxin for skeletal muscle nAChR, methyllycaconitine (MLA) for α7 or chimeric α7-5HT_3_, and epibatidine for α3β2 nAChR subtypes. As reported on Table 1, the 20-meSPX-G interacted less efficiently with the α4β2 nAChR subtype with a Ki = 3.6 nM.

## 3. Discussion

The toxicity of 20-meSPX-G was first tested using isolated mouse EDL nerve-muscle preparations revealing that the spirolide was able to block nerve-evoked muscle contractions in the nanomolar range (IC_50_ = 4.8 nM). Notably, concentrations of 20-meSPX-G that blocked nerve-evoked contractions had no action on directly-elicited contractions evoked by electrical stimulation, indicating a lack of direct effect on the contractile machinery of muscle fibers. These results suggested an action of the toxin at the neuromuscular junction rather than a direct action on skeletal muscle fibers. The comparison of potency of 20-meSPX-G with that of the well-known neuromuscular blocking agent *d*-tubocurarine [28], indicates that a 75-fold higher concentration of *d*-tubocurarine is needed to obtain a similar degree of neuromuscular blockade (IC_50_). Among cyclic imines, gymnodimine A (Figure 1D) was previously reported to block neuromuscular transmission in isolated mouse hemidiaphragm with an IC_50_ = 10.2 nM [20], whereas pinnatoxins E, F, and G blocked rat hemidiaphragm twitch-responses evoked by nerve stimulation with IC_50s_ ranging from 11 to 53 nM [29]. 

Further evidence that 20-meSPX-G is a potent blocker of neuromuscular transmission was obtained in vivo in anesthetized mice. The local injection of the spirolide caused a marked reduction of the CMAP amplitude evoked by nerve stimulation with an IC_50_ = 1.7 ng/kg, which is lower than that obtained with 13-desmethyl spirolide C (IC_50_ = 6 ng/kg) or gymnodimine A (IC_50_ = 1.6 µg/kg) [27]. Present results also disclosed that in vivo the action of 20-meSPX-G was fully reversible; such reversibility can explain the rapid mouse recovery with an absence of noticeable long-standing effects in mouse bioassays with doses of spirolides producing marked toxicity symptoms but no lethality [18,19]. 

The interaction of 20-meSPX-G with its molecular targets was studied using both voltage-clamp recordings, performed in oocytes having incorporated or expressing nAChRs, and in binding assays on cells expressing nAChR subtypes in their membranes. In those experiments it was clearly shown that in a concentration-dependent manner, 20-meSPX-G blocked ACh-evoked nicotinic currents, while it had no agonist action on the various nAChR studied. Thus, the order of potency of 20-meSPX-G, evaluated by voltage-clamp recordings (using IC_50_ values reported in the Results section, normalized to the ACh EC_50_ for each nAChR subtype) was as follows: α1_2_β1γδ (*Torpedo*) > α7 (human) > α4β2 (human). The order of antagonist potency of 20-meSPX-G, evaluated by comparing affinity constants (Ki) on binding assays (Table 1) was: α1_2_β1γδ (*Torpedo*) > α3β2 (human) > α7-5HT_3_ (chick) > α4β2 (human) nAChR. The affinity of 20-meSPX-G for the α1_2_β1γδ muscle-type nAChR was higher than that previously reported for other cyclic imine toxins such as 13-desmethyl spirolide C (Ki = 0.08 nM) > gymnodimine A (Ki = 0.23 nM) > pinnatoxin A (Ki = 2.8 nM) [20,21,24], and just about 2.5-fold lower (see Table 1) than that of the synthetic protein α-toxin from *Naja nigricollis*, a short-chain curare-mimetic toxin containing 61 amino acid residues and four disulfide bridges [30].

A major characteristic of neuronal nAChRs is their wide heterogeneity, based on both diverse subunit composition and stoichiometry [31,32]. In its interaction with the major neuronal nAChRs present in the central and peripheral nervous systems, 20-meSPX-G exhibited a broad specificity, and different potency on the subtype of receptors (Table 1). The highest affinity of 20-meSPX-G was observed on the human α3β2 nAChR, which was comparable to that of the epibatidine alkaloid, which is commonly used as a radiotracer because of its high affinity for some neuronal nAChR subtypes (Table 1). However, in contrast to epibatidine, 20-meSPX-G was unable to activate nAChRs by its own and, therefore, to exert an agonist action on nAChRs. Indeed, epibatidine, originally isolated from the skin of an Ecuadorian poisonous frog, (reviewed in [33]), is a potent agonist for the neuronal nAChRs [34,35]. 

Similar high affinity constants were determined on both human α7 and chick chimeric α7-5HT_3_ receptors. Some differences in the reported IC_50_ and Ki values for 20-meSPX-G on nAChRs were observed, which may be due to differences in the equilibrium conditions of the experiments (3 s for functional assays, and four hours for binding assays). It is worth noting that 20-meSPX-G was about 7.5-times more active than the diterpenoid plant alkaloid MLA considered to be a selective antagonist of the α7 subtype of nAChR [36]. Structural comparisons of the 13-desmethyl spirolide C complexes with the *Aplysia*-AChBP, as well as with MLA were previously reported [21]. From the structural point of view, 20-meSPX-G is closely related to 13-desmethyl spirolide C (Figure 1C), with a six-membered ring C bearing a hydroxyl group in the first case and a five-membered ring in the latter. The stereochemistry of the chiral center substituted by the hydroxyl group is currently unknown [12]. 

**Figure 7:**
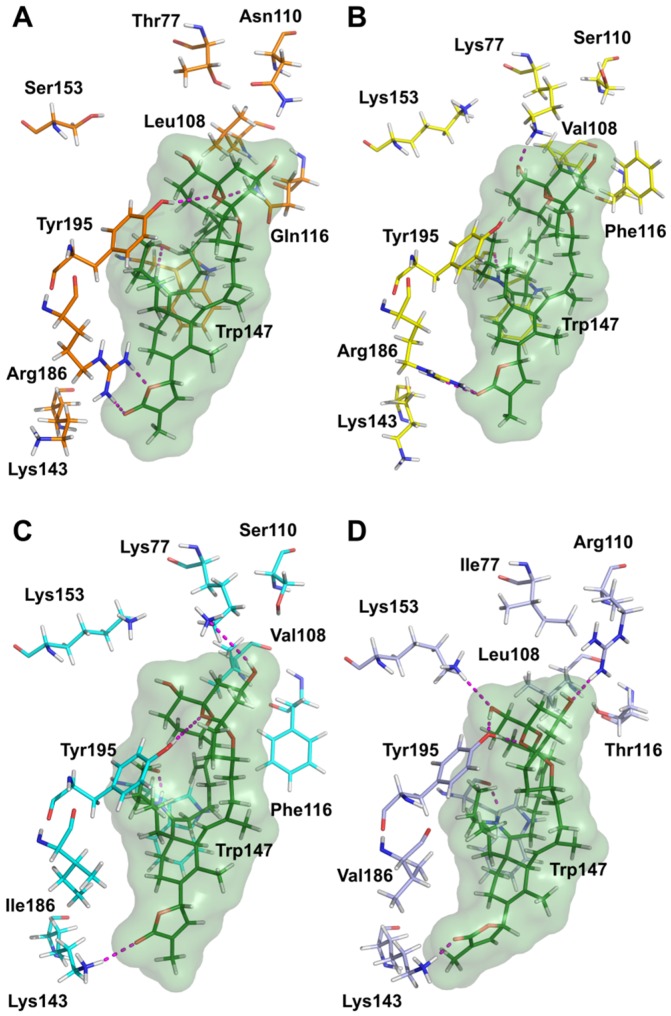
Protein-ligand interactions in the docking complexes of 20-meSPX-G (green) with four nAChR subtypes. (**A**) human α7 (orange, α7-α7 interface); (**B**) human α4β2 (yellow, α4-β2 interface); (**C**) human α3β2 (cyan, α3-β2 interface); and (**D**) *Torpedo* α1_2_β1γδ (light blue, α1-δ interface). Only amino acids interacting through hydrogen bonds with the ligand or involved in toxin’s subtype selectivity, and in the sequence alignment, are shown. The numbering of amino acid residues is the same as in [21].

Molecular docking calculations were performed to evaluate the interactions of 20-meSPX-G with the extracellular domain of the four nAChR subtypes studied (human α7, α4β2, α3β2, and α1_2_β1γδ). The receptor-ligand complexes (Figure 7) show binding modes similar to those observed in our previous studies, which were focused on the interaction between pinnatoxin A [24], 13-desmethyl spirolide C, and 13,19-didesmethyl spirolide C [23], and different nAChR subtypes. 

The position of the ligand is also analogous with the one reported in the crystal structure of the complex of 13-desmethyl spirolide C with the *Aplysia californica* AChBP [21]. All our docking complexes present a conserved hydrogen bond between the backbone C=O from Trp147 and the positively-charged spiroimine NH group. As already observed for 13-desmethyl spirolide C and 13,19-didesmethyl spirolide C [23], better interactions of 20-meSPX-G with α7 and α1_2_β1γδ nAChRs were evidenced, as compared with the α4β2 nAChR. They can be explained by the presence, in position 77, of Thr and Ile, respectively (relatively small amino acid residues), in the first case, which can provide accommodation to the spiroketal component, and in the position 108, the relatively large hydrophobic Leu residues which can through non-polar interactions stabilize the ligand. In contrast, the α4β2 nAChR subtype in position 77 is endowed with a Lys residue, whose size is not compatible with the presence of the spiroketal ring component in the binding site region, and the repositioning of this fragment towards the β2 subunit, which also makes impossible the hydrogen bond between this component and the side chain of Tyr195 that is present in all other cases.

The selectivity of 20-meSPX-G that was determined experimentally for α3β2 compared with α4β2 can be explained by the presence of an Ile residue in the position 186 of α3β2 (which can no longer establish a hydrogen bond interaction with the butyrolactone fragment) instead of Arg for α4β2. The overall consequence of this mutation is a small shift of the ligand in the binding site of α3β2, which enables a hydrogen bond between the side chain of Lys143 and the butyrolactone ring, and the recovery of the conserved interaction between the side chain of Tyr195 and the spiroketal ring system.

Our molecular docking results show that both stereoisomers are able to bind at the subunit interface, but only the one with the *S* stereochemistry can establish stabilizing interactions in some cases, through hydrogen bonds with Lys77 in α3β2 and with Arg110 in α1_2_β1γδ (Figure 7).

## 4. Conclusions

In conclusion, this study is the first to show that 20-meSPX-G in a concentration-dependent manner blocks mammalian neuromuscular transmission either in vitro or in vivo, and demonstrates that the toxin is a potent antagonist of postsynaptic muscle-types nAChRs. In addition, 20-meSPX-G blocked neuronal human α3β2 and chick chimeric α7-5HT_3_ with sub-nanomolar affinity, indicating that it is among the most potent cyclic imine toxins so far known. 

## 5. Materials and Methods

### 5.1. Reagents and Materials

20-methyl spirolide G (≥97% purity), obtained from laboratory cultures of the dinoflagellate *Alexandrium ostenfeldii*, was purchased from Cifga (Lugo, Spain). [^125^I]α−Bungarotoxin ([^125^I]α−BTX) (210–250 Ci·mmol−1), and (±)[^3^H]epibatidine (55 Ci·mmol^−1^), were purchased from PerkinElmer (Courtaboeuf, France). Isoflurane (AErrane^®^) was purchased from Baxter S.A. (Lessines, Belgium). Other chemicals were obtained from Sigma-Aldrich (Saint Quentin Fallavier, France), or other standard sources. The α-toxin from *Naja nigricollis* was synthesized, refolded, and purified, as previously described [30]. Methyllycaconitine (MLA), and (±)epibatidine were purchased from Tocris Bioscience (Bristol, UK).

### 5.2. Animals and Biological Materials

Adult female *Xenopus laevis* frogs were purchased at the Centre de Ressources Biologiques Xenopes—CNRS (Université de Rennes 1, Rennes, France), and *Torpedo marmorata* fish at the Service Modeles Biologiques of the Station Biologique de Roscoff (Roscoff, France). Swiss adult female mice were purchased at the Gif sur Yvette-CNRS animal house (CNRS, Gif sur Yvette, Essonne, France). All animal studies were performed in accordance with the guidelines established by the French Council on animal care “Guide for the Care and Use of Laboratory Animals”: EEC86/609 Council Directive—Decree 2001-131. The protocols were approved by the French Departmental Direction of Animal Protection (n° A91-453 to Evelyne Benoit) and the CNRS animal care and use committee.

The cDNA used in this work (coding for chick chimeric α7-5HT_3_, and human α4 and β2 subunits) were provided by Pierre-Jean Corringer (Pasteur Institute, Paris, France) and by Ortrud K. Steinlein (Institute of Human Genetics, Bonn, Germany). The human α7 cDNA was provided by Isabel Bermudez (Oxford Brookes University, Oxford, UK). 

All experiments were approved by the Animal Care and Use Committee of the French Ministry of National Education, High Education and Research (identification code: APAFIS#2671-2015110915123958 v3; date of approval: 27 November 2015).

### 5.3. Muscle Tension Measurements

Isometric twitch tension measurements were performed on isolated mouse EDL nerve-muscle preparations after rapid removal from euthanized animals. The isolated EDL nerve-muscle preparation was mounted in a silicone-lined bath perfused with a Krebs-Ringer solution of the following composition (in mM): NaCl 140, KCl 5, CaCl_2_ 2, MgCl_2_ 1, glucose 11, and HEPES 5 (pH 7.4), continuously perfused with pure O_2_ throughout the experiment. For muscle tension measurements, one of the muscle tendons was securely anchored onto the silicone-coated bath. The other tendon was attached to an isometric force displacement transducer (FT03, Grass Instruments, West Warwick, RI, USA) via an adjustable stainless-steel hook. Muscle twitches were induced either by motor nerve stimulation, or by direct electrical muscle stimulation with supramaximal 0.15 ms duration current pulses delivered by a stimulator (S-44 Grass Instruments), through an electrode assembly placed along the muscle length. The resting tension was monitored and kept constant for each tested preparation through a mobile micrometer stage. Signals from the force transducer were collected, and digitized with a Digidata-1322A A/D interface board (Axon Instruments, Union City, CA, USA), as previously reported [37]. 

### 5.4. Action on the Mouse Neuromuscular System in Vivo

In vivo recordings from the neuromuscular system were done in mice anesthetized by isoflurane inhalation, with a minimally-invasive electrophysiological method using Qtrac software (Institute of Neurology, Queen Square, London, UK, 2013), as previously described [27]. In brief, electrical stimulations were delivered at a constant frequency of 1.25 Hz to the caudal motor nerve (at the base of the tail) with surface electrodes, and the compound muscle action potential (CMAP) was recorded using needle-electrodes inserted into the tail muscle. 20-me-SPX-G, diluted in phosphate buffer saline containing <1% methanol, was injected intramuscularly (i.m.), between stimulation and ground electrodes, in a single bolus of 1.25−5 µL with a micro-syringe (10 μL volume). Continuous on-line recordings were started around six minutes before injections to determine the toxin and/or solvent effects, as a function of time, on selected excitability parameters, such as the CMAP amplitude and excitability threshold. 

### 5.5. Expression of Human α7 and α4β2 nAChRs in Xenopus Oocytes

Oocytes were obtained under anesthesia (ethyl-3-amino benzoate methanesulfonate salt solution (1 g·L^−1^, Sigma-Aldrich, Saint Quentin Fallavier, France) from mature female *Xenopus laevis* frogs. Oocytes were recovered and placed in a medium containing no added calcium and containing (in mM): NaCl, 88; KCl, 2.5; MgCl_2_, 1; and HEPES, 5 (pH 7.6). After extensive washing with this solution, oocytes were transferred to a Barth’s solution containing (in mM): NaCl, 88; KCl, 1; MgSO_4_, 0.33; CaCl_2_, 0.41; MgSO_4_, 0.82; Ca(NO3)_2_, 0.33; NaHCO_3_, 2.4; and HEPES, 10 (pH = 7.2) supplemented with kanamycine 0.1 mg·mL^−1^. Oocytes (stage V–VI) were defolliculated (manually) and microinjected with 50 nL of human α7 mRNA (0.1 μg·μL^−1^), or with a 50 nL mixture of plasmids carrying the cDNA of α4 and β2 (0.3 μg·μL^−1^, each). For micro-injection a Nanoliter 2000 Micro4 Controller (World Precision Instruments, Inc., Hertfordshire, UK) was used. Oocytes that were microinjected were incubated at 18 °C in Barth’s solution, and electrophysiological recordings were performed 3−4 days after microinjection. 

### 5.6. Microtransplantation of Torpedo nAChR to Xenopus Oocytes

*Torpedo marmorata* fish were anaesthetized with 0.03% tricaine (Sigma-Aldrich, Saint Quentin Fallavier, France) in seawater, before surgical removal of electric organs. From sliced *Torpedo* electric organs, purified membranes enriched in the α1_2_β1γδ nAChR were prepared at 4 °C in 5 mM glycine, using procedures described previously [21,38]. Aliquots of the purified membranes were stored at –80 °C until use. Microtransplantation of *Torpedo* nAChR [39] consisted in a single microinjection of a membrane suspension (50 nL at 3.5 mg·mL^−1^ protein) into the oocyte cytoplasm using a Nanoliter 2000 Micro4 Controller mounted on a microscope (World Precision Instruments, Inc., Hertfordshire, UK), as previously described [21].

### 5.7. Voltage-Clamp Recording on Oocytes

Currents evoked by ACh were recorded with a two-microelectrode voltage-clamp amplifier (OC-725B, Warner Instrument Corp., Hamden, CT, USA) at −60 mV holding potential. Micro-electrodes had 0.5–1.5 MΩ tip resistances. A pCLAMP-9/Digidata-1322A system (Molecular Devices, Union City, CA, USA) was used for data recording. The chamber for recordings had 300 μL capacity, and was superfused (8 mL·min^−1^, 20 °C) with a modified Ringer’s solution containing (in mM): NaCl, 100; KCl, 2.8; MgCl_2_, 1; BaCl_2_, 0.3; and HEPES, 5 (pH 7.4), where BaCl_2_ replacement to CaCl_2_ prevents secondary activation of Ca^2+^-dependent chloride current [39,40]. A computer-controlled solution-exchange system (VC-6, Warner Instruments Corp., Hamden, CT, USA) was used to superfuse ACh and 20-meSPX-G. In oocytes expressing the human α7 nAChR, ACh was perfused during 3 s periods, and for oocytes expressing the human α4β2 or having incorporated the *Torpedo* α1_2_β1γδ nAChR into their membrane, for 15 s periods. A 3 min interval was used between consecutive ACh applications, to guarantee nAChR recovery from desensitization. Dose-response inhibition curves were constructed and fitted to the equation *Y* = 100/(1 + 10^(Log IC_50_ − [*X*]) × *n*)^), where *Y* is the fractional (%) response remaining in the presence of inhibitor at concentration [X], IC_50_ is the inhibitor concentration that reduced the amplitude of ACh-evoked by 50% and n, is the Hill coefficient, using the software GraphPad Prism 6.05 (GraphPad Software, Inc., San Diego, CA, USA, 2013).

### 5.8. Expression of nAChRs in Human Embryonic Kidney Cells and Competition Binding Assays

The chimeric chick cDNA of the α7-5HT_3_ nAChR was transfected into human embryonic kidney (HEK-293) cells by calcium phosphate precipitation, as described [41,42]. Briefly, the cDNA (15 μg of α7-5HT3) was transfected by calcium precipitation with a careful control of pH (6.95). Cells were placed at 37 °C under 5% CO_2_, and 48 h after transfection were collected in a phosphate-buffered saline (PBS) with 5 mM Ethylene Diamine Tetraacetic Acid (EDTA), and suspended in 3 mL/plate of this buffer for binding experiments. For human α4β2 and α3β2 receptor subtypes, 24 h after the calcium phosphate transfection with neuronal nAChRs cDNA, the cells were placed for two days at 30 °C, 5% CO_2_ before collection for binding assays. Cell density was adjusted in order to specifically bind ≤10% of the radioligand.

#### Competition Binding Assays

Competition binding assays with *Torpedo* nAChRs were performed at equilibrium (incubation during 4 h with 0.05 μg of *Torpedo* membranes with different concentrations of 20-meSPX-G or α-toxin and [^125^I]α−BTX (0.25–0.45 nM). Nonspecific binding was performed in the presence of cobratoxin (1 μM). Filters washed with cold PBS were counted on a gamma counter (LKB-Multigamma 1261, Uppsala, Sweden). The affinity of 20-meSPX-G for the chimeric chick α7-5HT_3_ nAChR was determined as described previously [20,42]. [^3^H]epibatidine was used in equilibrium binding experiments with α4β2 and α3β2 nAChRs. Cells expressing these nAChRs were incubated with [^3^H]epibatidine (0.5−1 nM) and several concentrations of 20-meSPX-G or epibatidine for 4 h. After filtration, GF/C filters were dried and 6 mL of scintillation solution (Ultima Gold F, PerkinElmer, Courtaboeuf, France) were added before counting the filters on a Liquid Scintillation Analyzer (Tri-Carb 2300 TR, PerkinElmer, Inc., Waltham, MA, USA). The nonspecific binding was determined in the presence of epibatidine (100 nM). 

IC_50_ values were determined by fitting the competition data by the empirical Hill’s equation and converting to Ki constants using the equation: Ki = IC_50_/(1 + L*/Kd) [43]. The Kd for α-BTX (50 pM) on muscle-type receptor, and the Kd for epibatidine on human α3β2 and α4β2, was equal to 35 and 20 pM, respectively. All experiments were done at least three times in duplicate.

### 5.9. Molecular Modeling

Protocols used in the present study are similar to those defined for the interaction of pinnatoxin A, 13-desmethyl spirolide C, and 13,19-didesmethyl spirolide C with nAChRs [23,24]. Briefly, homology models for the extracellular domain of human α7, α4β2, α3β2 and *Torpedo* α1_2_β1γδ nAChRs subtypes were produced using the *Aplysia californica* AChBP crystal structure as a template (Protein Data Bank code 2WZY) [21]. Three-dimensional structures of the ligand (with the two possible configurations at C17 position bearing the hydroxyl group) were generated using Corina 3.6 (Molecular Networks GmbH, Erlangen, Germany, 2016). No docking software is presently able to deal natively with the flexibility of the macrocycle; therefore, the docking procedure was done in two steps: (i) conformational search of the stereoisomers was carried out using MacroModel (Schrödinger LLC, Portland, OR, USA) to produce 14 conformers for the stereoisomer *R* and 11 conformers for the stereoisomer *S*, incorporating the macrocycle flexibility; and (ii) molecular docking using Gold (Cambridge Crystallographic Data Centre, Cambridge, UK) of the two stereoisomers at the subunit interfaces of α7, α4β2, α3β2, and α12β1γδ homology models. The binding site, defined as a 15 Å radius sphere, was centered at half-distance between backbone oxygen atoms of residues Trp147 and Cys190 (using the GoldScore scoring function), all other parameters had default values. Side-chain flexibility was included at strategic binding site positions, whenever it was possible, to optimize the formation of protein-ligand interactions. Overall, the protocol that was used for the docking introduced flexibility at both the ligand (comprising the macrocycle) and the binding-site protein side chains, for generating credible ligand-protein complexes. The receptor-ligand complexes images were produced using PyMol (Schrödinger LLC, Portland, OR, USA).

## Figures and Tables

**Table 1:** Affinity constants of 20-meSPX-G (in nM) obtained in competition-binding assays at equilibrium, and the comparison with the snake α-toxin, methyllycaconitine (MLA) and epibatidine, toxic agents exhibiting high affinity for muscle and neuronal nAChRs, respectively.

Antagonist	α1_2_β1γδ (*Torpedo*)	α7-5HT_3_ (Chick)	α3β2 (Human)	α4β2 (Human)
20-meSPX-G	0.028 ± 0.005 ^a^	0.11 ± 0.08	0.040 ± 0.001	3.60 ± 0.07
α-Toxin (snake) ^b^	0.011 ± 0.002	-	-	-
MLA	-	0.83 ± 0.12	-	-
Epibatidine	-	-	0.034 ± 0.002	0.054 ± 0.011

^a^ Mean values (± SEM) from three distinct experiments performed in duplicate; ^b^ α-toxin from *Naja nigricollis*, a short-chain curaremimetic toxin (61 residues and four disulfides).
